# 高龄或脆弱弥漫大B细胞淋巴瘤诊治与全程管理中国专家共识（2025年版）

**DOI:** 10.3760/cma.j.cn121090-20250301-00104

**Published:** 2025-08

**Authors:** 中华医学会血液学分会淋巴细胞疾病学组 中国医药教育协会淋巴疾病专业委员会

## Abstract

随着人口老龄化，高龄弥漫大B细胞淋巴瘤（DLBCL）患者比例显著上升。老年综合评估是评价患者化疗耐受性的重要依据。高龄患者和老年综合评估为脆弱的DLBCL患者由于化疗耐受性差、合并症多，需进行个体化诊疗及全程管理。为提高我国临床医师对高龄或老年综合评估为脆弱的DLBCL患者的诊治水平，中华医学会血液学分会淋巴细胞疾病学组和中国医药教育协会淋巴疾病专业委员会组织相关专家制定了本共识。

## Abstract (en)

As the aging population increases, the proportion of elderly patients with diffuse large B-cell lymphoma（DLBCL）is significantly rising. Comprehensive geriatric assessment plays a crucial role in assessing chemotherapy tolerance. Very old or frail DLBCL patients classified by comprehensive geriatric assessment exhibit reduced tolerance to chemotherapy and multiple comorbidities, necessitating tailored treatment plans. To enhance the diagnostic and therapeutic capabilities of clinicians in China regarding very old or frail DLBCL patients classified by comprehensive geriatric assessment, the Lymphocyte Disease Group of the Hematology Division of the Chinese Medical Association and the Lymphatic Disease Society of the Chinese Medical Education Association have convened relevant experts to formulate this consensus.

弥漫大B细胞淋巴瘤（DLBCL）是最常见的淋巴瘤亚型，以老年人发病为主。根据2015年至2019年监测、流行病学和最终结果（surveillance, epidemiology and end results，SEER）数据库的研究数据，年龄≥80岁的DLBCL患者占总患者人数的20.0％[1]。随着中国人口老龄化的加剧，联合国人口基金预测，至2050年，中国80岁以上人口数量将从2020年的3 580万人增长至约1.47亿人[2]，因此≥80岁的DLBCL患者数量将会显著增加。高龄患者合并症多、化疗耐受性差，预后不良[3]。老年综合评估是评价老年DLBCL患者化疗耐受性的重要依据。中国老年DLBCL患者中应用意大利淋巴瘤基金会老年综合评估（FIL-CGA）系统评估为脆弱者占31.4％～35.9％[4]–[5]，2年总生存（OS）率约58.5％，2年无进展生存（PFS）率约37.3％[6]；中国老年DLBCL患者中应用北京医院创建的工具性日常生活活动能力（IADL）、年龄、合并症及血清白蛋白（IADL, age, combordities and albumin，IACA）指数评估系统评估为脆弱者约占16.2％，2年OS率为24.1％，2年PFS率为16.7％[7]。然而针对高龄或脆弱患者的临床研究较少，缺乏标准治疗方案且患者合并症及联合用药多，因此全程管理尤为重要。

为规范我国临床医师对高龄或脆弱DLBCL患者的诊治与全程管理，中华医学会血液学分会淋巴细胞疾病学组及中国医药教育协会淋巴疾病专业委员会组织相关专家根据国际与国内相关指南及循证医学证据，探讨并制定本共识，提出对中国高龄或脆弱DLBCL患者的诊治建议及全程管理方案。

一、高龄或脆弱患者的定义

高龄患者的年龄定义：依据《NCCN肿瘤临床实践指南：B细胞淋巴瘤2025.V2》和《中国临床肿瘤学会（CSCO）淋巴瘤诊疗指南（2024）》，本共识将高龄患者的年龄界定为≥80岁[8]–[9]。

脆弱患者的定义：依据老年综合评估进行界定，推荐应用以下老年综合评估系统：①IACA指数评估系统[7]，总分≥3分的患者，具体评分标准见表1。②FIL-CGA系统[5]，满足以下条件患者：日常生活活动能力（ADL）<5分，或IADL<6分，或改良老年疾病累计评分表（MCIRS-G）有≥1个3～4级合并症或2级合并症>8个，或年龄≥80岁的不适合组患者，具体见表2；③简化老年评估（sGA）系统[10]，年龄≥80岁且符合以下情况之一的患者：ADL<6分，或IADL<8分，或MCIRS-G有≥1个3～4级合并症或2级合并症≥5个的患者。

**表1:** IACA指数评估系统评分标准

指标	IACA评分（分）		
	0	1	2
IADL（分）	8	6～7	≤5
血清白蛋白（g/L）	≥34	<34	–
CCI（分）	<3	≥3	–
年龄（岁）	≤75	>75	–

**注** IADL：工具性日常生活活动能力；CCI：Charlson指数；–：无内容

**表2:** 意大利淋巴瘤基金会老年综合评估（FIL-CGA）分组标准

指标	适合组	不适合组	脆弱组
ADL（分）	6	5	<5
IADL（分）	8^a^	6～7^b^	<6^b^
MCIRS-G	无3～4级合并症且2级合并症<5个^a^	无3～4级合并症且2级合并症5～8个^b^	≥1个3～4级合并症或2级合并症>8个^b^
年龄	<80岁^a^	≥80岁的适合组患者^b^	≥80岁的不适合组患者^b^

**注** ADL：日常生活活动能力；IADL：工具性日常生活活动能力；MCIRS-G：改良老年疾病累计评分表；^a^同时满足；^b^或满足

二、诊断、预后评估与危险度分层

高龄或脆弱DLBCL患者诊断、鉴别诊断及分期参考国家卫生健康委员会发布的《弥漫性大B细胞淋巴瘤诊疗指南（2022年版）》。高龄DLBCL患者较年轻患者存在更多预后不良的生物学特征，如活化B细胞样（activated B-cell like，ABC）亚型、预后不良分子亚型比例较高[11]。上海交通大学医学院附属瑞金医院研究显示，MYD88^L265P^/CD79B突变占比与年龄呈正相关。MYD88^L265P^/CD79B突变可促进BCR/NFκB信号通路异常激活，为布鲁顿酪氨酸激酶抑制剂（BTKi）作为高龄DLBCL患者的治疗选择提供了理论基础[12]。鉴于分子生物学特征是评估患者预后、选择个体化治疗方案的重要参考依据，因此，推荐患者在条件允许情况下于诊断后进行二代测序等分子学检查。

除IPI、NCCN-IPI等常用的DLBCL危险度分层外，推荐对高龄或脆弱DLBCL患者使用老年预后指数（EPI）进行危险度分层[10]。EPI是针对老年DLBCL患者的特异性预后评分系统，纳入了sGA、IPI及血红蛋白作为指标：sGA适合组积0分、不适合组积3分、脆弱组积4分；IPI 1分积0分、IPI 2分积1分、IPI 3～5分积3分；血红蛋白≥120 g/L积0分、<120 g/L积1分。计算3项参数得分，总分0～1分为低危组、2～5分为中危组、6～8分为高危组。该评分系统可用于预测老年患者的OS率和早期死亡率，且已在中国人群中得到验证[13]。

三、治疗

对于高龄或脆弱DLBCL患者，治疗目的为改善症状，提高患者的生存质量，并尽可能使患者得到长期缓解，延长患者的OS期。选择治疗方案应综合考虑患者化疗耐受性、药物不良反应、合并症及联合用药、免疫和分子表型等因素，并结合患者意愿制定个体化的治疗方案。具体治疗方案选择见图1。

**图1:**
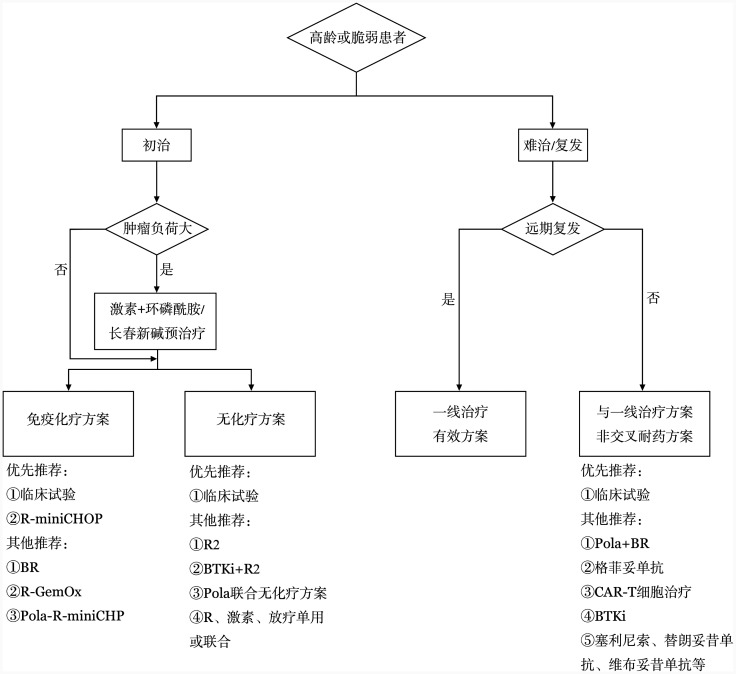
高龄或脆弱弥漫大B细胞淋巴瘤患者治疗流程图 **注** R-miniCHOP：利妥昔单抗+减量CHOP（环磷酰胺+多柔比星+长春新碱+泼尼松）；BR：苯达莫司汀+利妥昔单抗；R-GemOx：利妥昔单抗+吉西他滨+奥沙利铂；Pola-R-miniCHP：维泊妥珠单抗+利妥昔单抗+减量CHP（环磷酰胺+多柔比星+泼尼松）；R2：利妥昔单抗+来那度胺；BTKi：布鲁顿酪氨酸激酶抑制剂；Pola：维泊妥珠单抗；CAR-T细胞：嵌合抗原受体T细胞

（一）初治高龄或脆弱DLBCL

1. 免疫化疗方案

对于肿瘤负荷较大的患者，在免疫化疗前使用激素或激素联合长春新碱/环磷酰胺进行预治疗，待肿瘤负荷减轻后再进行免疫化疗。

（1）优先推荐：①临床试验。②R-miniCHOP［利妥昔单抗+减量CHOP（环磷酰胺+多柔比星+长春新碱+泼尼松）］方案：美国东部肿瘤协作组体能状态评分≤2分且无其他严重活动性疾病的高龄患者采用R-miniCHOP方案治疗，2年OS率可达到40.0％～70.0％，不良反应以骨髓抑制最常见[5],[14]，本共识作为优先推荐。

（2）其他推荐：①BR（苯达莫司汀+利妥昔单抗）方案：一项2期多中心临床试验中，BR方案治疗高龄或脆弱DLBCL患者，2年OS率和PFS率分别为51.0％和38.0％[15]，故可作为无法耐受R-miniCHOP方案患者的备选。②R-GemOx（利妥昔单抗+吉西他滨+奥沙利铂）方案：在年龄80岁及以上且其他器官无严重功能损害的亚组患者中，3年OS率和PFS率分别为67.0％和49.0％，推荐使用，尤其是无法耐受蒽环类药物的患者[16]。③维泊妥珠单抗（pola）-R-miniCHP方案：其在DLBCL的疗效已得到证实[17]。早期研究结果提示，pola-R-miniCHP方案在高龄或脆弱患者中，安全性与R-miniCHOP方案无显著差异[18]。因此，推荐在高龄或脆弱患者中进行尝试。

2. 无化疗方案

（1）优先推荐：临床试验。

（2）其他推荐：①R2（来那度胺+利妥昔单抗）方案：在一项多中心单臂2期临床研究中，R2方案治疗脆弱患者，2年OS率和PFS率分别为48.2％和40.5％，且耐受性较好[19]，可以作为脆弱患者的治疗选择。②BTKi+R2方案：针对75岁以上FIL-CGA评估为不适合或脆弱的患者，IR2（伊布替尼+R2）方案治疗的2年OS率和PFS率分别为66.7％和53.3％，不良反应可控[20]。新一代BTKi（如泽布替尼）联合R2方案的临床试验目前正在进行[21]–[22]，其安全性更高，推荐在高龄或脆弱患者中应用。对来那度胺不耐受患者也可尝试BTKi联合利妥昔单抗[23]。③pola联合无化疗方案：pola联合R2[24]、ZR（泽布替尼+利妥昔单抗）[25]、ZR2（泽布替尼+R2）[26]等方案在脆弱患者的早期研究中展示了其安全性和有效性，可作为高龄或脆弱患者的治疗选择。④其他：以上方案均无法耐受的患者，可选择利妥昔单抗、激素、放疗等单用或联合治疗作为姑息治疗。

（二）难治/复发的高龄或脆弱DLBCL

对于难治/复发的高龄或脆弱DLBCL患者，相关临床试验较少，应在难治/复发时再次评估患者疾病特征及耐受性等来制定个体化的治疗方案。远期复发患者可以使用一线治疗有效的方案，早期复发或原发难治患者可选择与一线治疗方案非交叉耐药的治疗方案。

1. 优先推荐：临床试验。

2. 其他推荐：①pola+BR方案：一项多中心随机对照临床研究表明，pola+BR方案治疗成人难治/复发DLBCL患者（33～86岁），2年OS率和PFS率分别为38.0％和28.4％，显著高于BR方案（2年OS率：17.0％；2年PFS率：9.1％），且未增加不良反应发生率[27]。推荐pola+BR方案作为高龄或脆弱难治/复发DLBCL患者的治疗选择。②格菲妥单抗（glofitamab）：格菲妥单抗在治疗成人难治/复发DLBCL患者（21～90岁）的关键2期临床试验中显示出良好疗效，老年患者与年轻患者完全缓解率差异无统计学意义（≥65岁患者对<65岁患者：38％对41％）[28]。中国多中心研究也验证了其在成人DLBCL患者（20～82岁）中的有效性和安全性，推荐用于高龄或脆弱患者[29]。③嵌合抗原受体T（CAR-T）细胞治疗：CAR-T细胞治疗是治疗成人难治/复发DLBCL的重要手段之一。真实世界研究表明，≥75岁的患者三线及以上接受CAR-T细胞治疗后OS与65～74岁的患者无显著差异[30]。国内的研究表明，不适合或脆弱患者接受CAR-T细胞治疗后不良反应发生率高于适合组患者[31]。因此，推荐CAR-T细胞治疗方案作为难治/复发高龄或脆弱DLBCL患者的三线及以上治疗选择，但应用时需临床医师进行综合评估和判断。④BTKi：既往治疗中未使用过BTKi的高龄或脆弱的难治/复发DLBCL患者，可尝试使用BTKi单药或联合治疗。⑤其他：塞利尼索[32]、替朗妥昔单抗（loncastuximab tesirine）[33]等药物，均推荐在难治/复发的高龄或脆弱DLBCL患者中进行尝试。对于CD30表达阳性的难治/复发的高龄或脆弱DLBCL患者，维布妥昔单抗[34]也可以进行尝试。

四、全程管理

由于高龄患者常合并心血管疾病、糖尿病、肝肾功能不全等基础疾病，其治疗耐受性显著降低。因此，在全程管理中，需依托多学科团队协作，开展全面综合评估、人群风险分层、个性化干预、持续随访监测的管理模式，对患者各系统合并症进行针对性管理。

（一）心血管疾病及心脏毒性管理

高龄或脆弱患者均属于肿瘤治疗相关心功能不全高危或极高危患者，治疗前应评估其心血管疾病及相关病史，完善相关辅助检查，对心血管疾病控制不佳或新发现可疑心血管疾病的患者，需请心血管疾病专科医师协助，全面评估心血管功能并予以相应治疗[35]。

在DLBCL治疗过程中，尽可能选用心脏毒性小的治疗方案，全程监测治疗方案的心血管毒性，同时有效控制心血管疾病危险因素。高龄或脆弱患者血压控制目标为<140/90 mmHg（1 mmHg＝0.133 kPa），耐受良好的患者血压应控制在130/80 mmHg以下。若治疗过程中出现急性心血管不良事件，应暂停治疗并请心血管专科医师协助诊治与评估。心血管疾病的管理及治疗、心血管毒性的监测应在治疗结束后持续进行。

（二）糖尿病与高血糖管理

高龄或脆弱患者糖尿病发病风险更高，治疗前应评估糖尿病及相关病史，完善相关辅助检查[36]。推荐高龄或脆弱合并糖尿病患者采取相对宽松的血糖管理目标：空腹血糖7.8～10.0 mmoL/L，餐后2 h或随机血糖10.0～13.9 mmol/L，糖化血红蛋白8.0％～9.0％。在DLBCL治疗过程中，对已确诊的糖尿病或高血糖患者进行持续血糖监测；可疑糖尿病或既往糖尿病血糖控制不佳的患者请内分泌专科医师协助诊治。此外，需警惕治疗过程中的应激事件引起的一过性血糖升高及高血糖相关急性事件（如酮症酸中毒、高渗性高血糖状态等），尤其是治疗相关高血糖。

（三）慢性肺病及呼吸道感染管理

推荐在初治时对高龄或脆弱患者肺部病变情况进行充分评估，包括慢性肺病及用药史、吸烟史等，处于慢性阻塞性肺疾病急性加重期的患者应通过胸部CT、肺功能等评估患者慢性肺病的严重程度，并给予相应的药物治疗和对症支持治疗。

年龄是药物相关间质性肺病（drug-induced interstitial lung disease，DILD）的高危因素。怀疑DILD的患者，应与肺部感染（尤其是卡氏肺孢菌感染）、淋巴瘤肺部进展等疾病相鉴别。确诊DILD后，根据病情严重程度进行分级诊疗，暂时或永久停用可疑药物，并给予糖皮质激素治疗和对症支持治疗[37]。必要时请呼吸科、影像科等科室协助诊治。

（四）消化系统疾病及消化系统不良反应管理

高龄或脆弱患者在治疗前应充分评估其消化系统疾病史并评估其基线肝功能、乙型肝炎病毒感染情况，谨慎使用糖皮质激素。

在DLBCL治疗过程中，应警惕药物性肝损伤（drug induced liver injury，DILI）的发生，治疗全程监测肝功能及凝血功能。DILI发生时应对其进行损伤程度分级，根据严重程度延迟、停用可疑药物，并采取其他治疗决策[38]。

（五）肾脏疾病及肾功能损伤管理

慢性肾脏病（chronic kidney disease，CKD）的发病率随年龄增长而逐渐增加，基础肾功能影响高龄或脆弱患者的治疗选择。初治时应详细询问肾脏病史并评估基础肾功能。对于合并CKD的患者，推荐通过患者的血清肌酐水平计算患者的估算肾小球滤过率（eGFR）并分级，结合患者年龄、性别、基础疾病、DLBCL分期等综合评估，确定是否需要调整治疗药物剂量，必要时可请肾内科、药剂科多学科诊疗[39]。全程动态监测患者的尿量以及血清肌酐等相关实验室指标变化，及早识别急性肾功能损伤，及时给予对症支持治疗，必要时暂停原发病治疗。

（六）自身免疫性疾病管理

对初治时合并自身免疫性疾病的患者，应全面评估疾病活动性、系统受累程度、治疗史、实验室指标等。DLBCL治疗药物（如利妥昔单抗、糖皮质激素）可能减弱自身免疫性疾病活动，但自身免疫性疾病治疗常用的免疫抑制剂会增加高龄或脆弱患者的感染风险。因此，这类患者应由专科医师联合诊治，根据自身免疫性疾病的病情变化及DLBCL治疗药物使用情况，动态调整治疗方案。

（七）神经系统疾病及神经系统损伤管理

高龄或脆弱患者为脑血管疾病的高发人群，治疗前应全面评估其脑血管病变史及危险因素、脑部病变及血管的基线情况。对存在脑血管病后遗症的患者，给予相应健康宣教和管理。在DLBCL治疗过程中发生急性脑血管事件的患者，应暂停原发病治疗，请神经内科、神经外科联合诊治，确定脑血管病治疗方案，待病情稳定后决定后续原发病治疗方案。怀疑新发脑血管病变应与原发病累及中枢神经系统相鉴别。

（八）营养管理

依据营养风险筛查2002（NRS 2002）量表评估，高龄或脆弱DLBCL患者均存在营养风险，需积极进行营养评估和干预[40]。

老年患者的能量目标为20～30 kcal·kg^−1^·d^−1^，肿瘤患者能量目标为25～30 kcal·kg^−1^·d^−1^，蛋白质摄入量为1.0～2.0 g·kg^−1^·d^−1^，同时结合患者体力活动水平、疾病状态和耐受性等因素进行个体化调整[40]。

根据患者具体情况确定营养支持途径，包括经口摄食、肠内营养及肠外营养。鼓励患者首先通过饮食调整来满足机体需求。若饮食量不能达到目标能量的60％持续3～5 d，建议补充口服营养，推荐能量为400～600 kcal/d。当肠内营养摄入量低于目标能量需要量的60％超过7 d，需考虑进行肠外营养支持治疗。

五、结语

高龄或脆弱DLBCL推荐应用老年综合评估识别脆弱患者，采用EPI作为患者的预后评估工具。针对高龄或脆弱DLBCL患者，推荐采用减量的化疗方案、无化疗方案或支持治疗等，并建议开展多中心、前瞻性的临床研究，以进一步挖掘适合高龄或脆弱患者的治疗方案。我们推荐加强对高龄或脆弱患者的全程管理，建立多学科合作的管理团队，对患者的合并症、药物不良反应及营养状态等进行全面、动态的综合评估，并监测DLBCL治疗药物的安全性，以优化治疗策略，实现个体化治疗，提升患者生活质量，延长生存时间。
